# Marion Harland

**Published:** 1904-02

**Authors:** 


### MARION HARLAND.

A very interesting feature of the Sunday edition of The Chicago Record-Herald, and one looked for by every woman reader of that paper, is the page devoted to Marion Harland. Under the heading "The Housewives' Exchange" queries and answers appear on subjects which conscientious housewives enjoy discussing. Wholesome advice is given about the care of children and how to make a home beautiful and attractive.

On the same page are also to be found some of Marion Harland's famous recipes. She is considered an authority on this subject, many people making it a practice to preserve her recipes whenever they are published.

A word about Marion Harland herself. Thousands of people who have read her articles are desirous of knowing more about her. Her real name is Mary Virginia Terhune. She was born in Amalia county, Virginia, Dec. 21, 1831; received a good education; began to write for the press at 14, and in 1856 married Rev. Edward Payson Terhune. Besides writing for the press, she is the author of a large number of books. She has a summer home called Sunnybank at Pompton, N. J., but at present is living in New York City.

